# In Vitro and In Vivo Biocompatibility of Boron/Nitrogen Co-Doped Carbon Nano-Onions

**DOI:** 10.3390/nano11113017

**Published:** 2021-11-10

**Authors:** Marta d’Amora, Adalberto Camisasca, Raul Arenal, Silvia Giordani

**Affiliations:** 1 Nano Carbon Materials, Istituto Italiano di Tecnologia (IIT), 16163 Genoa, Italy; marta.damora@iit.it; 2 School of Chemical Sciences, Dublin City University, Glasnevin, Dublin 9, Ireland; adalberto.camisasca@dcu.ie; 3 Instituto de Nanociencia y Materiales de Aragon (INMA), CSIC-U. de Zaragoza, 50009 Zaragoza, Spain; arenal@unizar.es; 4 Laboratorio de Microscopias Avanzadas (LMA), Universidad de Zaragoza, 50018 Zaragoza, Spain; 5 ARAID Foundation, 50018 Zaragoza, Spain

**Keywords:** carbon nano-onion, heteroatom doping, cells, zebrafish, biosafety

## Abstract

Boron/nitrogen, co-doped, carbon nano-onions (BN-CNOs) have recently shown great promise as catalysts for the oxygen reduction reaction, due to the improved electronic properties imparted by the dopant atoms; however, the interactions of BN-CNOs with biological systems have not yet been explored. In this study, we examined the toxicological profiles of BN-CNOs and oxidized BN-CNOs (oxi-BN-CNOs) in vitro in both healthy and cancer cell lines, as well as on the embryonic stages of zebrafish (*Danio rerio*) in vivo. The cell viabilities of both cell lines cells were not affected after treatment with different concentrations of both doped CNO derivatives. On the other hand, the analysis of BN-CNOs and oxidized BN-CNO interactions with zebrafish embryos did not report any kind of perturbations, in agreement with the in vitro results. Our results show that both doped CNO derivatives possess a high biocompatibility and biosafety in cells and more complex systems.

## 1. Introduction

Carbon nano-onions (CNOs) are a member of the carbon family consisting of a multi nested fullerene-like structure [1] and a size ranging from a few to tens of nm depending on the synthetic methodology [2]. In the last decade, CNOs have shown great promise in bio-related applications due to their small size, high surface area, and easy surface modification approaches, as well as low in vitro cytotoxicity [3,4] and excellent in vivo biocompatibility [4,5]. In particular, their remarkable emission properties, high cellular uptake, and low cytotoxicity of different fluorescent-labelled CNOs allowed their use as bioimaging probes in several cell lines [3,6,7]. In addition, compared to other fluorescent nanoparticles that require multi-step synthesis protocols, CNOs can be produced by the thermal annealing of detonation nanodiamonds, which ensure a low-cost, single-step, and high-yield way to achieve nanoparticle synthesis [8,9].

Furthermore, the high conductivity and chemical stability, together with the development of novel conjugation protocols with biologically relevant molecules, made this material very attractive for biosensing applications, showing an enhanced analytical performance in terms of sensitivity and limit of detection [10,11,12,13].

Recently, we reported a new member of the CNO family, namely a boron/nitrogen co-doped derivative (BN-CNOs), and demonstrated how the simultaneous introduction of both boron and nitrogen atoms was able to confer remarkable catalytic properties to the CNO materials towards the oxygen reduction reaction [14]. However, the biological behavior of this novel class of CNOs has not yet been explored. In addition, the presence of dopants, such as boron and nitrogen, may impact and change to some extent the biocompatibility of the CNOs. Considering that carbon nanomaterials employed in any application may result in human and environmental exposure [15,16,17], with a consequent potential risk of adverse effects, it is necessary to fully assess the toxicological profile of BN-CNOs. To this end, in this work, we evaluated for the first time the toxicity of BN-CNOs both in vitro and in vivo.

In addition, we decorated the CNO surface with oxygen-containing groups via an oxidation procedure in order to enhance the water solubility of the constructs, opening the way for further functionalization procedures with biologically relevant molecules.

BN-CNOs and their oxidized counterparts (oxi-BN-CNOs) were characterized by high-resolution transmission electron microscopy (HRTEM), electron energy loss spectroscopy (EELS), X-ray photoelectron spectroscopy (XPS), UV-Vis absorption spectroscopy, dynamic light scattering (DLS), attenuated total reflectance Fourier transform infrared spectroscopy (ATR-FTIR) and thermogravimetric analysis (TGA).

The potential in vitro cytotoxicity of the CNO materials was assessed on two different cell lines. For our investigation, we selected mouse embryonic fibroblast cells (NIH 3T3) and human breast adenocarcinoma cells (MCF7) as healthy and cancer cell lines, respectively. Even if these cellular screenings provided preliminary results on the nanotoxicity of the doped CNO derivatives, we decided to analyze in more detail their possible dose-dependent effects in vivo in zebrafish (*Danio rerio*).

Nowadays, zebrafish are a valuable and alternative animal model since their embryos and larvae are not considered animals until 5 days post fertilization (dpf), in accordance with the EU legislation [18]. Zebrafish are commonly used for nanotoxicity assessments due to their unique features, such as a short reproduction time, small size, and optical transparency, that allow for high-throughput assessments. Furthermore, they have a significant anatomical, physiological, and genomic homology to other higher vertebrates [19,20,21,22,23,24].

In line with previous studies, where zebrafish were successfully employed for evaluating the toxicity of several carbon nanomaterials [25], we explored the toxicological profile of the doped CNO derivatives in zebrafish (*Danio rerio*) using a 5-day embryonic assay. We analyzed the biological responses of both zebrafish embryos and developing larvae treated with BN-CNOs and oxi-BN-CNOs, in terms of mortality/survival rate, swimming (frequency of movements), cardiac (heart rate) activities, and hatching success.

## 2. Materials and Methods

### 2.1. Materials

All reagents and solvents were purchased from Merck (Dublin, Ireland). Detonation nanodiamonds (DNDs) were bought from Carbodeon Ltd. (Vantaa, Finland). All materials were used as received.

### 2.2. Material Synthesis and Characterization

Boron/nitrogen carbon nano-onions (BN-CNOs) were produced through a previously reported thermal annealing process utilizing DNDs and boric acid (H_3_BO_3_) as precursors [14]. The synthesis of oxidized BN-CNOs (oxi-BN-CNOs) was accomplished following a chemical oxidative procedure using 3 M nitric acid (HNO_3_) as oxidizing agent [3].

The local composition and the structure of the CNO materials were investigated via high-resolution transmission electron microscopy (HRTEM) (FEI-Thermo Fisher Scientific, Eindhoven, The Netherland). The HRTEM samples were prepared by dispersing a drop of the ultrasonicated suspension in ethanol onto carbon holey-supported Cu grids. We used two different aberrations-corrected FEI Titan microscopes, both operated at 80 kV. For HRTEM, a high-resolution, Cs-corrected FEI Titan-Cube was employed. Electron energy loss spectroscopy (EELS) measurements were developed using an FEI Titan Low-Base microscope (FEI-Thermo Fisher Scientific, Eindhoven, The Netherland) equipped with a Cs probe corrector, a monochromator, and ultra-bright X-FEG electron source. The convergence angle was 25 mrad, the energy resolution was ~1 eV and the convergence and collection angles were 25 and 50 mrad, respectively. XPS analyses were carried out on a PHI-5000 VersaProbe spectrometer (Physical Electronics Inc. (PHI), Chanhassen, MN, USA) equipped with a monochromatic Al Kα radiation source. XPS data processing was performed by using Multipak software (Version 9.7, Physical Electronics Inc. (PHI), Chanhassen, MN, USA) with energy calibration performed with respect to the C 1s peak. TGA analyses were performed on a Netzsch TG 209 F1 Libra thermogravimetric analyzer (NETZSCH-Gerätebau GmbH, Weimar, Germany) by heating up the samples and placing them in an Al_2_O_3_ pan from RT to 900 °C at 10 °C/min under a constant airflow. ATR-FTIR analyses were carried out on solid samples on a Perkin Elmer Spectrum 100 (Perkin Elmer, Dublin, Ireland) under air conditions in the 3000–700 cm^−1^ range. UV–Vis absorption spectra were acquired with a Shimadzu UV-1800 spectrophotometer (Shimadzu, Milton Keynes, UK) by using standard quartz cuvettes with a 10 mm path length. DLS analyses were carried out on a Malvern Nano-ZS instrument (Malvern Panalytical, Dublin, Ireland) operating in backscattering (173°) mode, with an automatic selection of the number of independent measurements and the optimal detector position. For absorption and DLS analyses, the CNO materials were dispersed via ultrasonication in deionized water at 1 mg/mL and then diluted to the desired final concentrations. Prior to the analyses, each solution was sonicated for an additional 10 min.

### 2.3. In Vitro Biological Methods

#### Cell Culture

Mouse embryonic fibroblast cells (NIH/3T3, ATCC CL173 passage number 3) and human breast adenocarcinoma cells (MCF7, ATCC HTB22, passage number 3) were cultured in Dulbecco’s modified Eagle’s medium (DMEM) (Thermo Fisher Scientific, Inc., Waltham, MA, USA), supplemented with 10% fetal bovine serum (FBS) (Thermo Fisher Scientific, Inc., Waltham, MA, USA), 100 IU/mL penicillin, and 100 μg/mL streptomycin (Thermo Fisher Scientific, Inc., Waltham, MA, USA) at 37 °C, in a humidified 5% CO_2_ atmosphere.

For the toxicity assessment, cells (1 × 10^4^ cells/well) were seeded in 96-well plates. Following overnight incubation, the medium was removed and replaced with fresh DMEM, containing various concentrations (0.5, 1, 5, and 10 μg/mL) of BN-CNOs and oxi-BN-CNOs for 72 h. Cells were incubated with 5% dimethyl sulfoxide (DMSO is toxic to any kind of cells) as a positive control, while cells with the fresh medium were used as a negative control. The cytotoxicity was assessed using WST-1 assay (Roche Applied Science, Mannheim, Germany), according to our previous report [4]. This simple colorimetric assay was based on the conversion of the tetrazolium salt WST-1 in a colored dye by cellular mitochondrial dehydrogenases enzymes. It measured the cells metabolic activity, which was proportional to the number of viable cells.

### 2.4. In Vivo Biological Methods

#### Husbandry and Toxicity Assessment

Adult zebrafish were maintained and treated as previously described [26]. Briefly, embryos at 4 h post fertilization (hpf) were gathered, washed several times with E3 medium, and exposed to different solutions of BN-CNOs or oxi-BN-CNOs (5, 10, 50, and 100 μg/mL) until 120 hpf. The possible effects of BN-CNOs and oxi-BN-CNOs on the embryonic developmental stages were assessed in terms of the heart rate, swimming activity, malformation rate, hatching rate, and survival rate using a stereomicroscope armed with a digital camera at different hours post fertilization. Each experiment was repeated three times. All animal experiments were performed in full compliance with the revised directive 2010/63/EU.

### 2.5. Statistical Analysis

All data were expressed as mean ± standard deviations of three replicates. Data were analyzed by one-way analysis of variance (ANOVA) in combination with Holm–Sidak post hoc test to compare each treated group with negative controls. A value of *p* ≤ 0.01 (marked by an asterisk) was considered statistically significant.

## 3. Results and Discussion

### 3.1. Synthesis and Characterization of the CNO Materials

**Figure 1:**
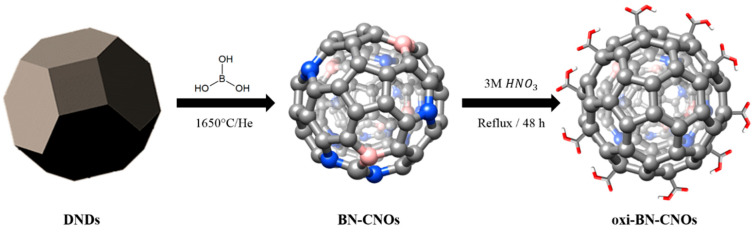
Synthetic procedure for the production of BN-CNOs and oxi-BN-CNOs.

The synthetic procedures employed for the production of boron/nitrogen doped carbon nano-onions (BN-CNOs) and its oxidized counterpart (oxi-BN-CNOs) are shown in Figure 1. The synthesis of BN-CNOs was accomplished by thermally annealing a mixture of DNDs and boric acid (H_3_BO_3_) at 1650 °C under a helium atmosphere [14], resulting in the efficient incorporation of the dopants into the carbon network. The modification of BN-CNOs via chemical oxidation to yield oxi-BN-CNOs was achieved by treating a CNO dispersion in 3 M nitric acid (HNO_3_) for 48 h under reflux conditions [3], resulting in a high coverage of the BN-CNO surface with carboxylic acid groups.

**Figure 2:**
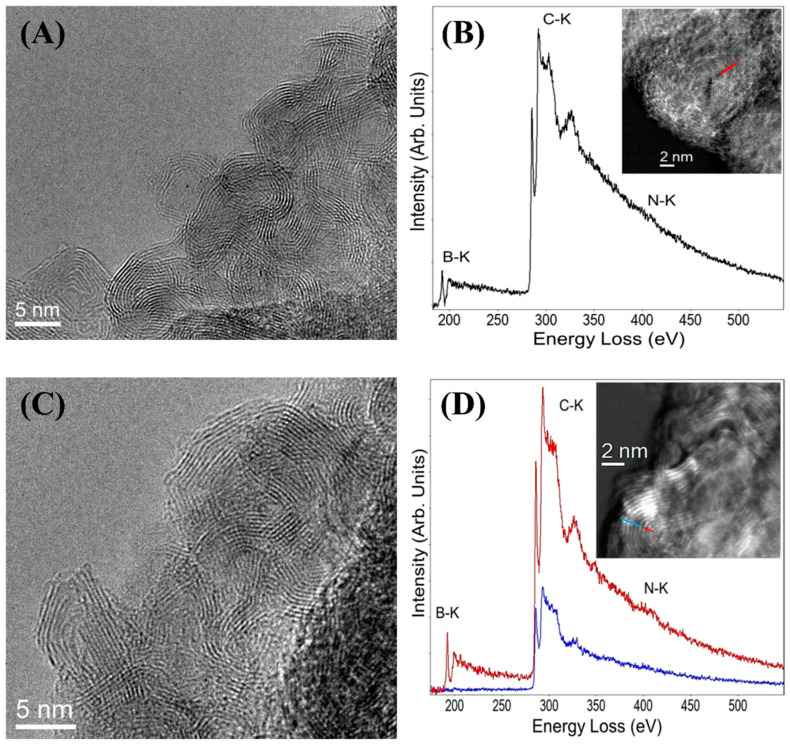
HRTEM images of BN-CNOs (**A**) and oxi-BN-CNOs (**C**), showing polyhedral-shaped CNOs. EELS spectra and corresponding STEM images as insets of BN-CNOs (**B**) and oxi-BN-CNOs (**D**), showing distinct B, C, and N–K edges. Blue and red lines in panel D refer to the external and internal layers, as depicted in the inset.

To cast light on the effects of the oxidation procedure, the morphological features of the CNO materials were investigated by HRTEM imaging. Figure 2A shows a representative HRTEM image of BN-CNOs, showing the existence of polyhedral CNOs as previously reported [14]. Following the oxidation procedure, no differences in the CNO morphology were observed (Figure 2C), suggesting that the process did not affect the integrity of the carbon structure.

EELS analyses were employed to obtain information on the chemical composition of the CNO derivatives. The EEL spectra of BN- and oxi-BN-CNOs are reported in Figure 2B,D, including the STEM images of the investigated areas for each sample, displayed as an insert. 

The EELS analysis of BN-CNOs (Figure 2B) reveals the presence of the boron (B), carbon (C), and nitrogen (N) K-edge structures. The distribution of these three elements indicates a mixture of both dopants in the carbon network, in line with what was previously reported [14]. Furthermore, the analysis of the fine structures near the edges (ELNES) for the K edges of B, C, and N revealed sp^2^-like phases, with a high crystalline quality [27,28,29,30,31].

In contrast, oxi-BN-CNOs exhibit a different behavior to that of BN-CNOs. Indeed, B, C, and N are only present in the innermost layers with the outmost layers showing only the C–K edge (red and blue lines in Figure 2D and its inset, respectively). This could be due to the surface removal of the dopants as a consequence of the oxidation process.

In any case, from the ELNES analyses of the three K edges, we can also confirm that both areas (internal and external) of the CNOs show a very good “graphitic-like” quality.

**Figure 3:**
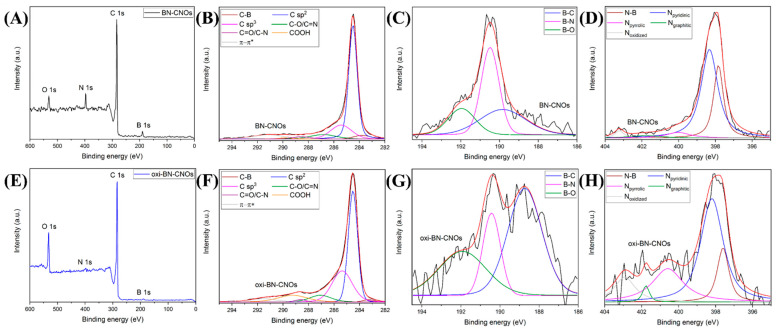
XPS survey (**A**,**E**) and high-resolution C 1s (**B**,**F**), B 1s (**C**,**G**), and N 1s (**D**,**H**) XPS spectra of BN-CNOs (upper panel) and oxi-BN-CNOs (lower panel), including peak deconvolution and experimental (black) and fitting curves (red).

The further confirmation of the removal of dopants from the CNO surface is provided by investigating the elemental composition of both samples by XPS. Figure 3A,E report the XPS survey spectra of BN-CNOs and their oxidized derivatives, displaying characteristic peaks due to the presence of carbon (C), boron (B), and nitrogen (N), as well as oxygen (O).

From the analysis of Table 1, it is clear that the oxidation procedure leads to a remarkable increase in the O content (from 4.6 to 12 at. %) of the oxidized CNO sample at the expense of B and N, whose content is drastically reduced (from 8 to 1.4 at. % and from 5.4 to 0.3 at. %, respectively), supporting the surface removal of the dopants as a consequence of the oxidation process. 

We report that the introduction of both dopants into the CNO structure increases the defectiveness of the carbon network [14]. As the presence of B and N atoms enhances the chemical reactivity of the doped structures; therefore, they likely represent preferential sites for oxidation [32,33] and this can explain their lower content in the oxidized CNOs. In agreement with the EELS results, the modification of the CNO surface during the oxidation likely removes B and N from the external layers of the materials (with the latter to a great extent compared to the former) with the creation of sp^3^ hybridized carbon atoms. On the contrary, as the oxidation occurs preferentially on the CNO surface, the inner part is not affected by the procedure, showing a preserved doped nature.

Further information regarding the chemical states of the elements can be obtained from the analysis of the high-resolution C 1s, B 1s, and N 1s XPS spectra of BN- and oxi-BN-CNOs, reported in Figure 3. Each spectrum, after a post-processing peak deconvolution process, is composed of different contributions reported in Table 2 and Table 3.

The C 1s peak of BN-CNOs (Figure 3B) consists of seven different components [14]. The peak at 283.7 eV is assigned to the C-B bonding state [34], while the two most intense peaks located at 284.5 and 285.4 eV are attributed to carbon atoms with sp^2^ and sp^3^ hybridization, respectively. The other components at higher binding energies are ascribed to oxygen- and nitrogen-bonded carbon atoms [35,36] (C–O and C=N at 286.8 eV, C=O, and C–N at 288.5 eV, as well as COOH at 289.8 eV) and the π−π* contribution (291.1 eV). After the oxidation, the analysis of the C 1s spectrum of oxi-BN-CNOs, reported in Figure 3F, reveals a consistent increase in the peak corresponding to the COOH content, and thus confirming the preferential formation of the carboxylic acid functionalities onto the CNO surface. Moreover, the substantial enhancement of the sp^3^ character, together with the decrease in the nitrogen- and boron-bonded carbon states, is in agreement with the above-discussed EELS results. In addition, the intensity decrease in the C=C bonds and π−π* contributions upon oxidation, which is ascribed to the photo-ionization of the π-electrons in conjugated systems [37], is in line with the introduction of defects into the graphitic nanostructures.

Additional insights on the effects of the oxidation on the chemical environment can be deduced by studying the differences between the B 1s and N 1s peaks of BN-CNOs (Figure 3C,D) and oxi-BN-CNOs (Figure 3G,H). The B 1s peak can be deconvoluted into three components assigned to B-C, B-N, and B-O bonding states [14,38,39], which are located at 189.8, 190.5, and 191.9 eV, respectively. While in the pristine material the B-N species are predominantly present, the oxidation leads to a drastic reduction of these bonding states (i.e., more than half of the total content), suggesting a preferential consumption in favor of the carbon- and oxygen-bonded states (Table 3). Similar results can be observed from the analysis of the N 1s peak, which is composed of five different contributions located at 397.8, 398.3, 400.0, 401.8, and 403.2 eV, ascribed to N-B, pyridinic, pyrrolic, graphitic, and oxidized N types, respectively [14,39,40]. After the oxidation, in line with what was observed for the B 1s peak, a marked decrease in the N-B bonding states is detected upon oxidation together with a minor reduction of the pyridine and graphitic N atoms (Table 3). Therefore, the increase in the pyrrolic-like and oxidized N species strongly suggests that their formation is probably due to the oxidation of the former types of N atoms, as reported for other N-doped carbon nanostructures [33,41].

Therefore, from the XPS analyses, it can be deduced that the oxidation preferentially disrupts the B-N species, which are the predominant bond type in BN-CNOs, and this accounts for the marked decrease in the B and N contents in the oxidized counterparts.

UV-Vis absorption spectroscopy and DLS were employed to investigate the effects of the oxidation procedure on the water dispersibility of oxi-BN-CNOs. 

**Figure 4:**
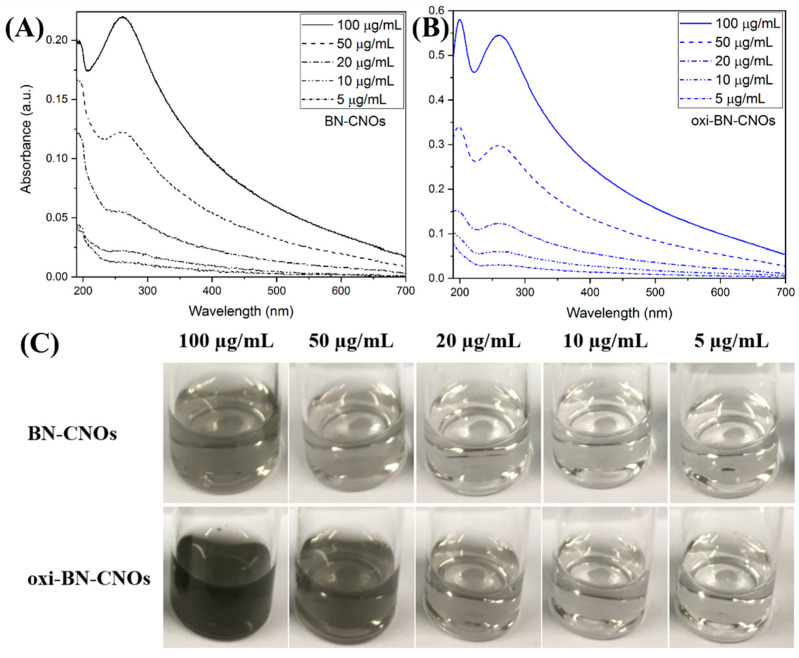
UV-Vis absorption spectra of (**A**) BN- and (**B**) oxi-BN-CNO dispersions in deionized water at different concentrations (5, 10, 20, 50 and 100 µg/mL). (**C**) Pictures of the solutions at the different concentrations.

Figure 4A,B show the UV-Vis spectra of BN- and oxi-BN-CNO dispersions in deionized water at different concentrations (5–100 μg/mL). Both samples exhibit absorption over the whole wavelength range with two absorption maxima, attributed to the π→π* electronic transition of the C=C bonds, located approx. at 200 and 260 nm. Compared to that of BN-CNOs, the oxidized material displays much higher absorbance values at all investigated concentrations, thus suggesting a better water dispersibility of the oxidized derivatives ascribed to the increased hydrophilicity imparted by the introduction of carboxylic acid groups onto the material surface by the oxidative treatment.

Further evidence of the superior dispersion abilities of oxi-BN-CNOs is provided by the analysis of the CNO solutions reported in Figure 4C, showing darker colors for the oxidized CNO at the same concentration (bottom panel in Figure 4C), and thus confirming that the introduction of carboxylic acid groups onto the material surface provides an excellent water dispersibility to the oxidized derivatives.

**Figure 5:**
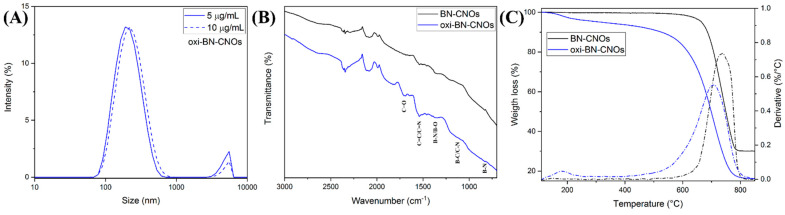
(**A**) DLS spectra of oxi-BN-CNO dispersions in deionized water at concentrations of 5 and 10 μg/mL. (**B**) FTIR spectra and (**C**) TGA (solid lines) and corresponding weight loss derivatives (dotted lines) of BN—(black) and oxi-BN-CNOs (blue).

Additional information about the dispersing abilities of both samples was obtained by DLS. Figure 5A shows the DLS spectra of the oxi-BN-CNO water dispersions at two representative concentrations (5 and 10 μg/mL), showing the hydrodynamic diameters of 216.2 and 223.3 nm, respectively. Although we were able to obtain the UV-Vis absorption spectra for BN-CNOs (Figure 4A), their dispersions were not homogeneous enough for assessing reliable DLS measurements due to the polydisperse nature of the particles caused by the heavy aggregation of the materials in water. On the contrary, as depicted in Table 4, it is evident that the oxidation treatment imparts a notable dispersing effect on oxi-BN-CNOs, as indicated by the hydrodynamic size values around 220 nm and negligible differences in the concentrations, thus confirming the superior dispersion in water to that of BN-CNOs.

Further confirmation of the successful surface modification of the co-doped CNOs was obtained by FTIR analyses, providing additional information regarding the functional groups introduced during the CNO surface modification (Figure 5B). 

While the FTIR spectrum of BN-CNOs, reported in black in Figure 5B, displays typical peaks assigned to mixed B, C, and N bonding states [14], an additional peak at around 1700 cm^−1^ is observed in the oxi-BN-CNO FTIR spectrum (blue line in Figure 5B), which is attributed to the carbonyl group (C=O) stretching vibration modes. Therefore, the appearance of this new contribution confirms the formation of carboxylic acid functionalities over the CNO surface, in perfect agreement with what was observed from the analysis of the high-resolution C 1s XPS spectrum of oxi-BN-CNOs (Figure 3F).

Further confirmation of the successful surface modification of the co-doped CNOs was obtained by FTIR and TGA analyses. The analysis of the weight loss and derivative curves of both CNO materials, which is shown in Figure 5B, reveals that BN-CNOs exhibit a remarkable thermal stability up to 600 °C, when the decomposition of the carbon network starts occurring [14]. On the other hand, the oxidized CNOs exhibit a two-stage decomposition behavior. The first mass loss, occurring below 200 °C, is due to the degradation of the oxygen-containing groups, while the second is due to the decomposition of the carbon structure. The net weight loss at 450 °C (approx. 7%) and the decreased decomposition temperature (approx. 30 °C less than that of BN-CNOs), as shown in Table 4, accounts for the introduction of defects as a consequence of the oxidation process, thus confirming the successful functionalization of the CNO surface. In addition, the lower mass residue at 850 °C reported for the oxidized derivate, attributed to the generation of boron oxides [14,42], further confirms the consumption of boron induced by the oxidative process.

Our characterizations confirm the successful formation of the oxidized doped CNOs, thus allowing us to investigate the biological behavior of these new members of the CNO family both in vitro and in vivo.

### 3.2. In Vitro and In Vivo Studies

To accurately define the cytotoxicity of the CNO materials on cells, we decided to employ both a healthy (mouse embryonic fibroblast cells, NIH 3T3) and a cancer cell line (human breast adenocarcinoma cells, MCF7). The possible effects of both CNOs on NIH 3T3 and MCF7 cells were assessed by means of the colorimetric WST1 assay. The two cell lines were incubated with BN- and oxi-BN-CNOs for 24, 48, and 72 h.

**Figure 6:**
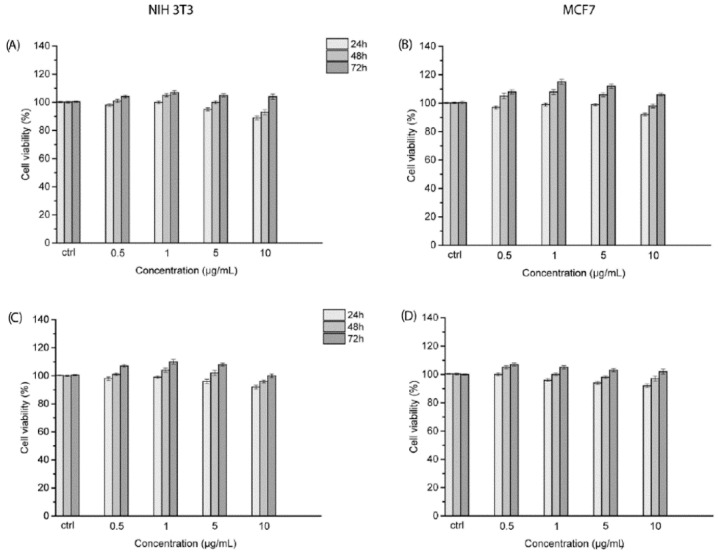
Cellular viability of NIH 3T3 and MCF7 cells treated with various concentrations of BN- (**A**,**B**) and oxi-BN-CNOs (**C**,**D**) for 24, 48, and 72 h. Data are expressed as mean ± standard deviations of three replicates.

Figure 6 displays the cell viability of NIH 3T3 and MCF7 cells after the exposure at the same concentrations (0.5, 1, 5, and 10 μg/mL) of the CNO materials. 

The cell viabilities of NIH 3T3 and MCF7 cells were not affected after the treatment with the different dilutions of BN-CNOs (Figure 6A,B). NIH 3T3 and MCF7cells treated with the highest dose of BN-CNOs showed cell viability values of 90% and 92%, respectively, after 24 h of incubation. With a similar behavior, no inhibitory effects were observed upon NIH 3T3 and MCF7 cells exposed to oxi-BN-CNOs (Figure 6C,D) for all the treatments (0.5, 1, 5 and 10 μg/mL) and time points. NIH 3T3 and MCF7 cells treated with the highest dose of oxi-BN-CNOs showed cell viability values of 92% and 93%, respectively, after 48 h of incubation. These results show a high cyto-biocompatibility of BN- and oxi-BN-CNOs in both healthy and cancer cells. 

These findings are in agreement with our previous in vitro reports on the toxicological profile of CNOs. In fact, we previously reported a low toxicity or absence of toxicity of different CNO derivatives in different cell lines, including KB cells, HeLa Kyoto cells, human breast carcinoma cells (MDA-MB-231), and human ovarian carcinoma cells (A2780 cells) [2]. However, the newly reported doped CNOs seem to exhibit a higher biocompatibility with respect to the previously reported CNO materials. In particular, they present values of cell viabilities 5 to 10% higher than those shown by the other CNO derivatives at the highest concentrations tested (i.e., 5 and 10 μg/mL).

To corroborate the toxicological profile in the cells, the effects of the doped CNOs in zebrafish during the growth were assessed by treating fertilized embryos in solutions of BN-CNOs and oxi-CNOs at different dilutions (5, 10, 50, and 100 μg/mL). 

**Figure 7:**
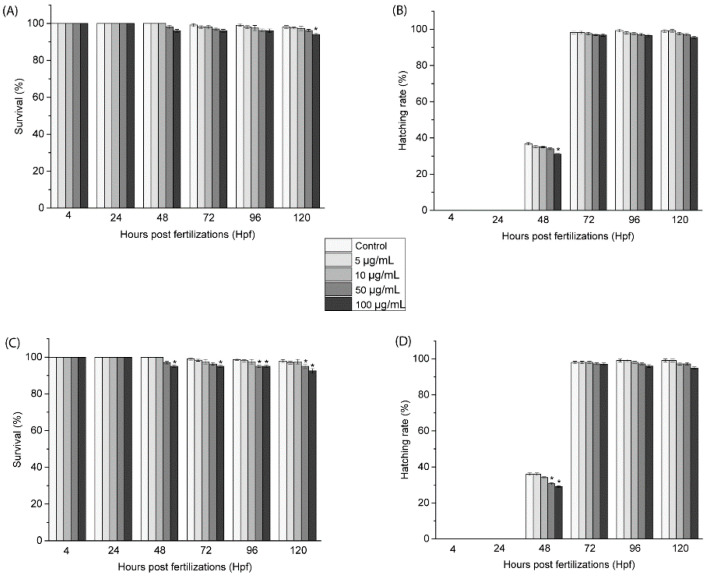
Survival rates (**A**–**C**) and hatching rates (**B**–**D**) of embryos exposed to different concentrations of BN- and oxi-BN-CNOs (5, 10, 50, and 100 μg/mL). Data are calculated as means ± S.D., from three independent experiments, *n* = 80 (* indicate *p* ≤ 0.01 compared to the control).

Several toxicological endpoints were evaluated during the whole treatment (4–120 hpf), as reported in Figure 7. The survival rates of zebrafish embryos exposed to BN-CNOs, shown in Figure 7A, presented no significant changes in comparison to the control groups up to 50 μg/mL, while they induced a significant change at 100 μg/mL after 120 hpf. On the other hand, the survival rates of zebrafish embryos exposed to oxi-BN-CNOs (Figure 7C) showed important changes only between 96 and 120 hpf at a dose of 50 μg/mL and between 48 and 120 hpf at a concentration of 100 μg/mL. Similarly, the time of hatching was not affected by the exposure to BN-(Figure 7B) and oxi-BN-CNOs (Figure 7D), showing no inhibition in this parameter. These values of survival and hatching rates indicated a nontoxic profile of both materials, as per OECD guidelines (normative law). 

**Figure 8:**
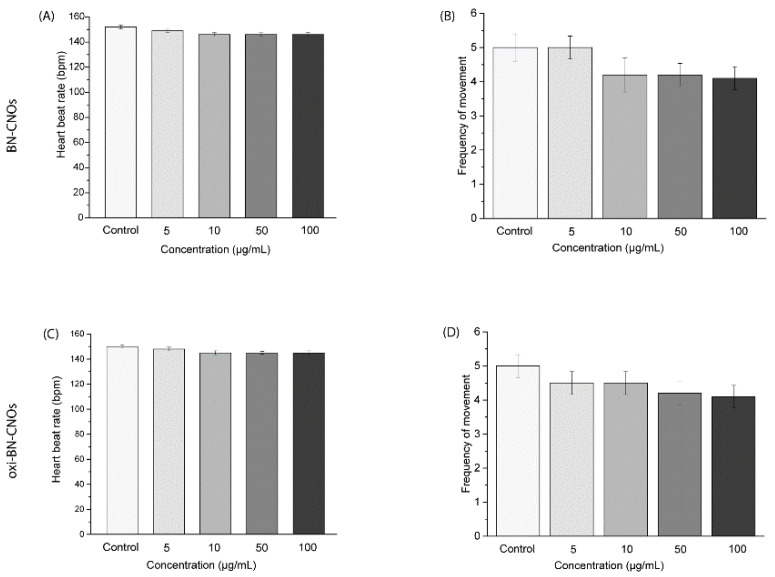
Heart rate (**A**–**C**) and frequency of voluntary movements (**B**–**D**) of larvae at 72 hpf exposed to different concentrations of BN- and oxi-BN-CNOs.

In addition, we evaluated the heart rate of the exposed larvae, as shown in Figure 8. The average value of heartbeats showed no considerable changes compared to the controls (Figure 8A,C). Additionally, both BN-CNOs and oxi-BN-CNOs did not exert an influence on the swimming activity of treated larvae, with no consequent reduction/increase in the movement frequency with respect to the controls (Figure 8B,D).

The values of these toxicological endpoints demonstrated the biosafety of the CNO materials in the range of the investigated parameters. These findings are in agreement with the in vitro assessment in NIH 3T3 and MCF7 cells and with previously reported studies on the biocompatibility of different CNO derivatives in *Danio rerio* during the growth [5].

## 4. Conclusions

In this work, we evaluated for the first time the toxicological profile of a novel class of CNO materials, namely BN- and oxi-BN-CNOs. Our characterization techniques showed that the reported doping approach is effective in the introduction of both dopants onto the CNO surface, while the oxidation procedure is able to decorate the CNO surface with oxygen functionalities at the expense of the heteroatoms. This is attributed to the enhanced reactivity provided by the dopants that act as preferential sites.

Our in vitro, performed on both healthy and cancer cell lines, and in vivo studies confirm the low cytotoxicity and excellent biocompatibility of these novel CNO derivatives, opening the way for the exploration and use of the doped CNOs in biological applications.

## Figures and Tables

**Table 1:** Elemental compositions of BN- and oxi-BN-CNOs from XPS analyses.

Sample	C (at. %)	O (at. %)	B (at. %)	N (at. %)
BN-CNOs	82.0	4.6	8.0	5.4
oxi-BN-CNOs	86.2	12.0	1.4	0.3

**Table 2:** Chemical state, positions (eV), and relative area percentages of the deconvoluted C 1s peaks obtained from XPS analyses of BN- and oxi-BN-CNOs.

Sample	C-B	C sp^2^	C sp^3^	C–O/C=N	C=O/C–N	COOH	π−π*
BN-CNOs	283.7	284.5	285.4	286.8	288.6	289.8	291.1
	(3.4%)	(59.6%)	(17.4%)	(7.1%)	(2.5%)	(0.9%)	(9.1%)
oxi-BN-CNOs	283.3	284.5	285.4	287.0	288.6	289.3	291.5
	(1.2%)	(46.5%)	(29.6%)	(7.2%)	(1.2%)	(9.8%)	(4.5%)

**Table 3:** Chemical state, positions (eV), and relative area percentages of the deconvoluted B 1s and N 1s peaks obtained from XPS analyses of BN- and oxi-BN-CNOs.

Sample	B–C	B–N	B–O	N–B	N_pyridinic_	N_pyrrolic_	N_graphitic_	N_oxidized_
BN-CNOs	189.8	190.5	191.9	397.8	398.3	400.0	401.8	403.2
	(33.8%)	(45.3%)	(20.9%)	(28.4%)	(59.5%)	(5.9%)	(3.4%)	(2.8%)
oxi-BN-CNOs	188.7	190.4	191.9	397.6	398.2	400.6	401.8	402.9
	(51.2%)	(19.45%)	(29.4%)	(13.4%)	(49.1%)	(23.3%)	(2.4%)	(11.8%)

**Table 4:** Hydrodynamic diameters of oxi-BN-CNOs at 5 and 10 µg/mL in deionized water from DLS analyses and weight loss (w.l.) at 450 °C, decomposition temperatures and residues at 850 °C of BN- and oxi-BN-CNOs from TGA analyses.

Sample	Size @ 5 µg/mL	Size @ 10 µg/mL	w.l. @ 450 °C	T_D_	Residue @ 850 °C
BN-CNOs	-	-	0.3%	736.0 °C	30.1%
oxi-BN-CNOs	216.2 ± 12 nm	223.3 ± 8 nm	7.3%	706.0 °C	16.1%

## Data Availability

The data presented in this study are available on request from the corresponding author.
